# Predictive impact of soluble interleukin‐2 receptor and number of extranodal sites for identification of patients at very high risk of CNS relapse in diffuse large B‐cell lymphoma

**DOI:** 10.1002/jha2.393

**Published:** 2022-02-08

**Authors:** Takafumi Shichijo, Hiro Tatetsu, Kisato Nosaka, Yusuke Higuchi, Yoshitaka Kikukawa, Yoshitaka Inoue, Kosuke Toyoda, Jun‐ichirou Yasunaga, Masao Matsuoka

**Affiliations:** ^1^ Department of Hematology, Rheumatology and Infectious Diseases Kumamoto University Hospital Kumamoto Japan; ^2^ Department of Hematology and Oncology Kumamoto City Hospital Kumamoto Japan

**Keywords:** CNS relapse, CNS‐IPI, diffuse large B‐cell lymphoma, extranodal site, sIL‐2R

## Abstract

There remains an unmet clinical need to identify which patients with diffuse large B‐cell lymphoma (DLBCL) would benefit from central nervous system (CNS) prophylaxis, due to the low positive predictive value (PPV; 10%–15%) of the currently available predictive models. To stratify patients at high risk of developing CNS relapse, we retrospectively analyzed 182 patients with DLBCL initially treated with rituximab, cyclophosphamide, doxorubicin, vincristine, and prednisone (R‐CHOP), or a R‐CHOP‐like regimen. Among them, 17 patients relapsed with CNS involvement, and the 2‐year rate of CNS relapse was 7.9%. Upon carrying out multivariate analysis, ≥3 extranodal sites and elevated soluble interleukin‐2 receptor (sIL‐2R) levels at diagnosis were identified as independent risk factors for CNS relapse. The 2‐year and 3.5‐year rates of CNS relapse were 57.1% and 78.6%, respectively, in patients with both elevated sIL‐2R and ≥3 extranodal sites. Furthermore, combined use of these risk factors of both elevated sIL‐2R and ≥3 extranodal sites resulted in a high PPV (71.4%), negative predictive value (93.1%), and overall accuracy (92.3%) for undergoing CNS relapse. In conclusion, we propose a simple and valuable tool to predict patients with DLBCL at very high risk of CNS relapse.

## 1 INTRODUCTION

Diffuse large B‐cell lymphoma (DLBCL) is the most common subtype of lymphoma, which represents almost 30% of non‐Hodgkin lymphoma cases [1]. Although approximately 60% of patients can be cured with rituximab, cyclophosphamide, doxorubicin, vincristine, and prednisone (R‐CHOP) or similar regimens, outcomes for remaining patients with relapse, or refractory disease is unfavorable [1]. The locations of relapse in most patients are nodal or extranodal sites, without central nervous system (CNS) lesions. However, approximately 3%–5% of patients develop CNS relapse [2, 3, 4]. One reason for CNS relapse could be attributed to the inability of R‐CHOP to cross the blood–brain barrier, resulting in inefficient concentrations involving CNS regions [5]. Compared to relapse without involvement of the CNS region, CNS relapse is a devastating complication with a short median overall survival (OS) of <6 months [6].

Several models have been reported to identify patients at high risk of CNS relapse [7, 8, 9, 10, 11, 12]. The German High‐Grade non‐Hodgkin Lymphoma Study Group (DSHNHL) proposed a model known as the CNS‐international prognostic index (IPI) to estimate risk of CNS relapse, which comprises five IPI factors and involvement of the kidney or adrenal glands [8, 13]. Another model indicated that the higher the number of extranodal sites assessed using positron emission tomography/computed tomography (PET/CT) imaging, the more frequent the incidence of CNS relapse [11]. In addition, certain specific extranodal localizations, such as the testis, kidney, and adrenal gland, are known to be risk factors for CNS relapse [14, 15, 16, 17]. These models or risk factors provide us with the ability to predict CNS relapse at 2 years by up to 10%–26.4%. In general, CNS prophylaxis is recommended for high‐risk patients. Importantly, however, more than half of high‐risk patients based on current models do not develop CNS relapse, resulting in these patients receiving unnecessary CNS prophylaxis [7, 8, 9, 10, 11, 14, 15, 16, 17, 18]. Therefore, a better predictive model to identify patients at very high risk of CNS relapse is highly desirable. Furthermore, the establishment of the optimum CNS prophylaxis is warranted to reduce the risk of CNS relapse in DLBCL.

The most useful prognostic indices for DLBCL patients are revised IPI and National Comprehensive Cancer Network IPI [13, 19, 20]. In addition, levels of soluble interleukin‐2 receptor (sIL‐2R), which is expressed on the surface of lymphocytes and is activated only in mononuclear cells such as T cells, B cells, monocytes, and natural killer cells, have been reported to be a prognostic factor in DLBCL [21, 22, 23, 24]. However, it remains unclear whether serum sIL‐2R levels are associated with CNS relapse in patients with DLBCL.

In this study, we retrospectively evaluated the risk factors for CNS recurrence in DLBCL patients who were treated with R‐CHOP or R‐CHOP‐like regimens, in order to identify patients at high risk of CNS relapse. In addition, we propose a predictive tool for CNS recurrence, which includes combined assessment of sIL‐2R levels and the number of extranodal sites involved.

## 2 METHODS

### 2.1 Study design

This was a single‐center, retrospective analysis of patients who were initially diagnosed as having DLBCL [25, 26] and treated with R‐CHOP(‐like) therapies at Kumamoto University Hospital between April 2010 and September 2020. Patients with CNS involvement at initial diagnosis, primary mediastinal B‐cell lymphoma, intravascular large B‐cell lymphoma, HIV‐1 infection, and transformation from indolent lymphoma were excluded.

Using this cohort, we investigated clinical characteristics at diagnosis and evaluated the incidence and risk of CNS relapse. Clinical data for each patient were extracted from medical records.

This study was approved by the institutional review board of Kumamoto University Hospital and was conducted in accordance with the principles of the Declaration of Helsinki.

### 2.2 Involvement of extranodal site and CNS regions

Involvement sites were defined based on the Lugano 2014 criteria [27]. The tumor mass was diagnosed using 18F‐fluorodeoxyglucose PET/CT. Involvement sites were determined based on diagnostic reports provided by radiologists.

CNS relapse was defined as DLBCL that relapsed within the brain parenchyma, spinal cord, leptomeninges, or eyeball upon CNS imaging or cerebrospinal fluid (CSF) evaluation. Patients predicted to be at high risk of CNS relapse (i.e., high CNS‐IPI score; involvement of the kidney/adrenal glands, testis; sites of anatomic proximity to CNS; positive for CD5 expression) were subjected to CSF evaluation and CNS prophylaxis (intrathecal [IT] chemotherapy of 15‐mg methotrexate [MTX] + 40 mg cytarabine + 20 mg prednisolone) upon the treating physicians’ decision.

### 2.3 Measurement of sIL‐2R

Serum sIL‐2R levels were routinely measured in all patients with DLBCL at diagnosis using chemiluminescent enzyme immunoassay (CLIA; STACIA, LSI Medience, or Determiner CL IL‐2R, Kyowa Medex CO., Ltd).

### 2.4 Statistical analysis

The probability of OS and rate of CNS relapse were calculated using the Kaplan–Meier method, and the groups were compared using the log‐rank test. OS was defined as the duration from initial diagnosis to death from any cause, or the date of the last follow‐up. The time to CNS relapse was defined as the duration from initial diagnosis to the first CNS relapse. We used the Cox proportional hazards model to evaluate risk factors for CNS relapse. Variables with *p*‐value < 0.10 by univariate analysis were included in a multivariate Cox proportional hazards model. Receiver operating characteristic (ROC) curve analysis was used to assess the cut‐off values for sIL‐2R levels. The cut‐off value was determined using the Youden index. Overall accuracy was calculated as follow: (true positive + true negative)/total number x 100. Statistical significance was defined as a two‐sided *p*‐value <0.05. Statistical analyses were performed using the EZR software package, v.1.54 (Saitama Medical Center, Jichi Medical University, Saitama, Japan), which is a graphical user interface for R (The R Foundation for Statistical Computing, v.3.6.2) [28].

## 3 RESULTS

### 3.1 Patient characteristics

**FIGURE 1:**
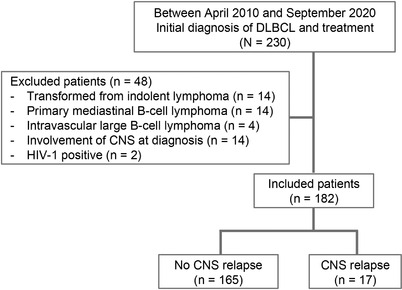
Consort diagram. CNS, central nervous system; DLBCL, diffuse large B‐cell lymphoma; HIV, human immunodeficiency virus.

During the study period, 230 patients were initially diagnosed as having DLBCL and treated at the Kumamoto University Hospital. Among them, patients diagnosed as having transformation from indolent lymphoma (*n* = 14), primary mediastinal B‐cell lymphoma (*n* = 14), and intravascular large B‐cell lymphoma (*n* = 4) were excluded. In addition, CNS involvement at diagnosis (*n* = 14) and human immunodeficiency virus (HIV)‐1‐infected patients (*n* = 2) were excluded. This resulted in a total of 182 patients with DLBCL, who were then considered as subjects for analysis (Figure 1). With a median follow‐up of 33.9 months (range, 1.61–134.8 months), 17 of these patients relapsed with CNS involvement.

Clinical characteristics of the 182 patients with DLBCL are listed in Table 1. Their median age was 70.6 years (range, 34.6–88.3 years), and 139 patients (76.4%) were aged >60 years old. According to the CNS‐IPI score, 48 patients (26.4%) were classified into the high‐risk group.

**TABLE 1:** Patient characteristics

		All (*n* = 182) *n* (%)
Age, median (range), years	70.6 (34.6−88.3)	
	<60	43 (23.6)
	≥60	139 (76.4)
Sex		
	Female	77 (42.3)
	Male	106 (57.7)
ECOG performance status		
	0−1	144 (79.1)
	2−4	38 (20.9)
Ann Arbor stage		
	I−II	75 (41.2)
	III−IV	106 (58.8)
LDH, median (range), IU/L	258 (125−2225)	
	≤ULN	72 (39.6)
	>ULN	127 (60.4)
Extranodal site(s)^a^		
	0	51 (28.0)
	1	78 (42.9)
	2	36 (19.8)
	≥3	17 (9.3)
CD5		
	No	134 (73.6)
	Yes	23 (13.7)
	Missing	25 (13.7)
sIL‐2R, median (range), U/ml	1033 (195−90,840)	
	Nonelevated (<3912)	147 (80.8)
	Elevated (≥3912)	35 (19.2)
IPI		
	Low risk (0−1)	46 (25.3)
	Low‐intermediate risk (2)	46 (25.3)
	High‐intermediate risk (3)	45 (24.7)
	High risk (4−5)	45 (24.7)
CNS‐IPI		
	Low risk (0−1)	46 (25.3)
	Intermediate risk (2−3)	88 (48.4)
	High risk (4−6)	48 (26.4)
Years of treatment		
	2010−2015	83 (45.6)
	2016−2021	99 (54.4)
Frontline immunochemotherapy		
	R‐CHOP	106 (58.2)
	R‐THP‐COP	66 (36.3)
	R‐CVP	3 (1.6)
	R‐CHOEP	2 (1.1)
	DA‐EPOCH‐R	5 (2.7)
CNS prophylaxis		
	No	150 (82.4)
	Yes (intrathecal alone)	32 (17.6)

Abbreviations: CHOEP, cyclophosphamide, doxorubicin, vincristine, etoposide, and prednisone; CHOP, cyclophosphamide, doxorubicin, vincristine, and prednisone; CNS, central nervous system; CVP, cyclophosphamide, vincristine, and prednisone; DA‐EPOCH, dose‐adjusted etoposide, prednisone, vincristine, cyclophosphamide and doxorubicin; ECOG, Eastern Cooperative Oncology Group; IPI, international prognostic index; LDH, lactate dehydrogenase; R, rituximab; sIL‐2R, soluble interleukin‐2 receptor; THP‐COP, pirarubicin‐ cyclophosphamide, vincristine, and prednisone; ULN, upper limit of normal.

^a^ Extranodal sites were defined based on the Lugano 2014 criteria.

### 3.2 Incidence and risk of CNS relapse

**FIGURE 2:**
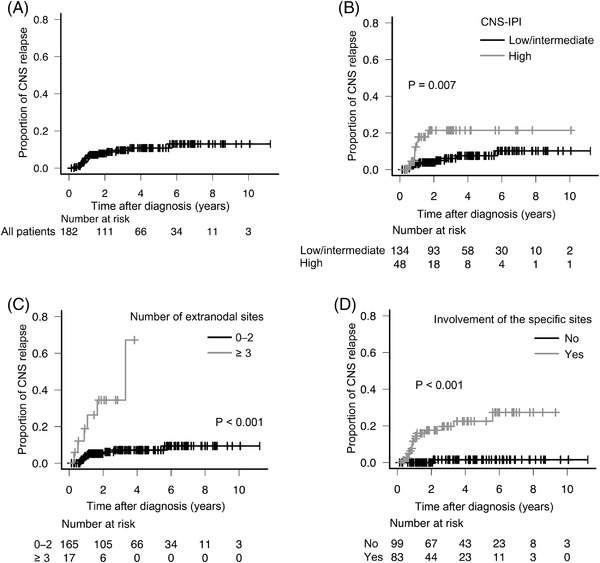
Incidence and risk of CNS relapse. Probability of CNS relapse in 182 patients with DLBCL (A); in patients with high versus low/intermediate risk according to CNS‐IPI (B), and the number of extranodal sites (0–2 vs. 3 or more) (C); and in patients with or without involvement of specific sites (D). CNS, central nervous system; CNS‐IPI, central nervous system international prognostic index; DLBCL, diffuse large B‐cell lymphoma

The median time to CNS relapse from diagnosis was 323 days (range, 106–2,049 days). As shown in Figure 2A, the 2‐year rate of CNS relapse in 182 patients with DLBCL was 7.9% (95% confidence interval [CI], 4.6–13.2). According to the CNS‐IPI score, the 2‐year rates of CNS relapse were 3.9% (95% CI, 1.6–9.1) and 21.4% (95% CI, 11.2–38.7) in patients with low/intermediate risk and high risk, respectively (*p* = 0.007, Figure 2B). Furthermore, the 2‐year rates of CNS relapse were 5.2% (95% CI, 2.7–10.2) and 34.5% (95% CI, 15.8–64.7) in patients with 0–2 and ≥3 extranodal sites, respectively (*p* < 0.001, Figure 2C).

Since the number of extranodal sites was linked to the incidence of CNS relapse, we evaluated sites of tumor involvement, including extranodal and several nodal sites (e.g., the tonsils, Waldeyer's ring, and spleen). On univariate analysis, involvement of the kidney/adrenal gland, testis, bone/bone marrow, liver, paranasal sinus, and spleen was identified as risk factors for CNS relapse (Table 2). On multivariate analysis, involvement of the kidney/adrenal gland, testis, bone/bone marrow, paranasal sinus, and spleen was associated with CNS relapse (Table 2). The 2‐year rates of CNS relapse in patients who had involvement at any of these five sites and those who had none were 17.6% (95% CI, 10.6–28.5) and 0.0%, respectively (*p* < 0.001; Figure 2D).

**TABLE 2:** Univariate and multivariate analyses of tumor‐involvement sites as the risk factors for developing CNS relapse

		Univariate analyses		Multivariate analyses	
	Number of patients, *n* (%)	HR (95% CI)	*p‐*Value	HR (95% CI)	*p‐*Value
Kidney/adrenal gland	13 (7.1)	5.62 (1.82−17.33)	0.003	4.27 (1.25−14.58)	0.020
Stomach	12 (6.6)	0.94 (0.13−7.12)	0.956	–	–
Small intestine	13 (7.1)	1.77 (0.40−7.75)	0.448	–	–
Testis	11 (10.4) ^a^	3.38 (0.97−11.78)	0.056	6.36 (1.61−25.19)	0.008
Uterus	3 (3.9) ^a^	5.42 (0.70−42.06)	0.106	–	–
Bone/bone marrow	50 (27.5)	4.46 (1.69−11.74)	0.002	3.02 (1.08−8.47)	0.034
Liver	6 (3.3)	4.95 (1.12−21.85)	0.035	–	–
Lung	14 (7.7)	2.75 (0.79−9.59)	0.113	–	–
Muscle	39 (21.4)	0.86 (0.25−2.99)	0.808	–	–
Orbit	5 (2.7)	2.21 (0.29−16.67)	0.444	–	–
Paranasal sinus	24 (13.2)	3.37 (1.18−9.63)	0.023	3.12 (1.02−9.59)	0.047
Thyroid	8 (4.4)	1.22 (0.16−9.17)	0.849	–	–
Spleen	17 (9.3)	4.41 (1.55−12.54)	0.005	4.83 (1.51−15.43)	0.008

*Note*: Factors with *p *< 0.1 by univariate analyses were included in the multivariate analyses.

There were no CNS relapse events in patients with involvement of the eyelids, gingiva, palate, Waldeyer's ring, salivary glands, esophagus,

duodenum, colon, peritoneum, subcutaneous, breast, fallopian tubes, ovaries, prostate, and ureter.

Abbreviation: CI, confidence interval; CNS, central nervous system; HR, hazard ratio.

^a^ Percentage of patients with testis or uterus involvement relative to the male and female cohorts, respectively.

### 3.3 Identification of patients at very high risk of developing CNS relapse

**FIGURE 3:**
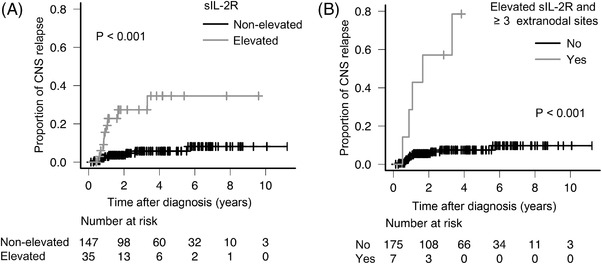
Association of incidence of CNS relapse with serum sIL‐2R levels. Probability of CNS relapse in patients with elevated (≥3912 U/ml) versus nonelevated (<3912 U/ml) sIL‐2R levels (A), and in patients with elevated sIL‐2R levels, according to the number of extranodal sites (0–2 vs. 3 or more) (B). CNS, central nervous system; sIL‐2R, soluble interleukin‐2 receptor

As several studies have reported that elevated sIL‐2R level is a useful marker for poor prognosis of DLBCL [21, 22, 23, 24], we evaluated the association of sIL‐2R abundance with the incidence of CNS relapse. Median sIL‐2R levels were 1024 U/ml (range, 195–90,840 U/ml) in the no CNS relapse group, and 3921 U/ml (range, 319–40,888 U/ml) in the CNS relapse group. In addition, ROC curve analysis indicated that the cut‐off value of sIL‐2R levels that distinguished CNS relapse from no CNS relapse was 3921 U/ml (area under the ROC curve, 0.639 [95% Cl, 0.485–0.793]; specificity, 84.2%; sensitivity, 52.9%; Figure S1). The 2‐year rate of CNS relapse in the elevated sIL‐2R level group (≥3921, *n* = 35) was higher than that in the nonelevated sIL‐2R level group (<3921, *n* = 147) (27.3% [95% CI, 14.5–47.8] and 3.6% [95% CI, 1.5–8.5]; *p* < 0.001, Figure 3A). Upon focusing on the risk factors for developing CNS relapse, other than tumor involvement sites, high score of CNS‐IPI, ≥3 extranodal sites, and elevated sIL‐2R levels were found to be statistically significant by univariate analysis (Table 3). Multivariate analysis showed that ≥3 extranodal sites and elevated sIL‐2R levels were independent risk factors for developing CNS relapse (Table 3). Consequently, in patients with both elevated sIL‐2R levels and ≥3 extranodal sites, the 2‐year and 3.5‐year rates of CNS relapse were 57.1% (95% CI, 26.6–90.2) and 78.6% (95% CI, 41.4–98.8) (*p* < 0.001; Figure 3B). Furthermore, the risk factors comprising both elevated sIL‐2R levels and ≥3 extranodal sites produced a higher positive predictive value (PPV), negative predictive value (NPV), and overall accuracy of 71.4% (95% CI, 29.0–96.3), 93.1% (95% CI, 88.3–96.4), and 92.3% (95% CI, 87.4–95.7), respectively, for developing CNS relapse, compared to high score of CNS‐IPI, ≥3 extranodal sites, elevated sIL‐2R, and specific involvement sites (the kidney/adrenal gland, testis, bone/bone marrow, paranasal sinus, and spleen) (Table 4).

**TABLE 3:** Univariate and multivariate analyses of risk factors for CNS relapse

	Univariate analyses		Multivariate analyses	
	HR (95% CI)	*p‐*Value	HR (95% CI)	*p‐*Value
CNS‐IPI				
Low/Intermediate	Reference		–	
High	3.43 (1.31−8.95)	0.012	–	–
Extranodal site(s)				
0−2	Reference		Reference	
≥3	8.10 (2.91−22.53)	<0.001	4.48 (1.49−13.42)	0.007
CD5				
No	Reference		–	
Yes	1.39 (0.39−4.88)	0.610	–	–
sIL‐2R				
Nonelevated	Reference		Reference	
Elevated	6.41 (2.44−16.83)	<0.001	4.31 (1.51−12.31)	0.006

*Note*: Factors with *p* < 0.1 in univariate analyses were included in the multivariate analyses.

Abbreviations: CI, confidence interval; CNS, central nervous system; IPI, international prognostic index; sIL‐2R, soluble interleukin‐2 receptor; HR, hazard ratio.

**TABLE 4:** Positive predictive value, negative predictive value, and overall accuracy in the prediction of CNS relapse

	PPV (95% CI)	NPV (95% CI)	Overall accuracy (95% CI)
CNS‐IPI high risk	16.7% (7.5−30.2)	93.3% (87.6−96.9)	73.1% (66.0−79.4)
≥3 Extranodal sites	35.3% (14.2−61.7)	93.3% (88.4−96.6)	87.9% (82.3−92.3)
Specific involvement site ^a^	19.3% (11.4−29.4)	99.0% (94.5−100.0)	62.6% (55.2−69.7)
Elevated sIL‐2R	25.7% (12.5−43.3)	94.6% (89.6−97.6)	81.3% (74.9−86.7)
Elevated sIL‐2R and ≥3 extranodal sites	71.4% (29.0−96.3)	93.1% (88.3−96.4)	92.3% (87.4−95.7)

Abbreviations: CI, confidence interval; CNS, central nervous system; IPI, international prognostic index; NPV, negative predictive value; PPV, positive predictive value; sIL‐2R, soluble interleukin‐2 receptor.

^a^ Involvement of the kidney/adrenal gland, testis, bone/bone marrow, paranasal sinus, and spleen.

### 3.4 Intrathecal chemotherapy for prophylaxis of CNS relapse

Among patients identified with a high risk of developing CNS relapse (those with ≥3 extranodal sites, elevated sIL‐2R levels, high risk of CNS‐IPI, and involvement of high‐risk organs, namely the kidney/adrenal gland, testis, bone/bone marrow, paranasal sinus, and spleen), there were no statistically significant differences in the rate of CNS relapse between patients with CNS prophylaxis and those without it (Table S1). In the CNS relapse group (*n* = 17), the median time to CNS relapse from diagnosis was 416 days (range, 254–1523 days) and 317 days (range, 106–1212 days) in patients with and without CNS prophylaxis, respectively (*p* = 0.161). However, the probability of 5‐year OS was 26.8% (95% CI, 1.3–67.0) in patients with CNS prophylaxis (*n* = 7) and 37.5% (95% CI, 9.9–65.9) in patients without CNS prophylaxis (*n* = 10) (*p* = 0.436; Figure S2).

## 4 DISCUSSION

Our study demonstrated that assessment of the number of extranodal sites in combination with sIL‐2R levels is a potentially useful tool to identify DLBCL patients at very high risk of CNS relapse. In addition, our real‐world data analysis identified that high risk of CNS‐IPI, ≥3 extranodal sites, and involvement of certain specific extranodal sites represented risk factors for CNS relapse.

Several studies have indicated that sIL‐2R level is a valuable marker for categorizing DLBCL patients with unfavorable outcomes [21, 22, 23, 24]. Although the tumor microenvironment has been reported to possibly contribute to increased serum concentrations of sIL‐2R, it remains unresolved why elevated sIL‐2R levels are associated with poor prognosis in patients with DLBCL [29]. Our finding that high levels of sIL‐2R were an independent predictive marker for CNS relapse suggested its role as a prognostic marker. Thus, therapeutic strategies to prevent CNS relapse in patients with elevated sIL‐2R levels may improve outcomes in this cohort.

Schmitz et al. developed CNS‐IPI, which is the most clinically utilized model for predicting CNS relapse in DLBCL. According to this model, the 2‐year CNS relapse rates in the low‐, intermediate‐, and high‐risk groups are 0.6%, 3.4%, and 10.2%, respectively [8]. This provides a robust and readily calculable estimation of risk for patients with DLBCL that has been validated in another independent cohort with similar results [11]. Therefore, CNS‐IPI has been adopted in a number of guidelines and recommendations [30, 31, 32, 33]. Our real‐world data also verified that CNS‐IPI predicted the risk of CNS relapse, despite a higher percentage of patients with high CNS‐IPI scores (26.4%) in our study than those in the DSHNHL cohort (12.2%) [8]. The reason of high population of patients with high CNS‐IPI score in our cohort is mainly due to high number of patients with old age and poor performance status (PS): our cohort versus DSHNHL cohort (aged >60 years old, 76.4% vs. 45%; poor PS, 20.9% vs. 11%). Since it is well known that Japan is one of the largest geriatric populations in the world, our cohort may reflect this trend. Furthermore, the large patient cohort with high CNS‐IPI scores may be one of the reasons for the overall high rate of CNS relapse in this study. Another predictive model of CNS relapse that is routinely available in clinical practice involves the increased number of extranodal sites [11]. The 3‐year cumulative incidence of CNS relapse is high (15.2%) in patients with ≥3 extranodal sites [11]. In this study, the absolute number of extranodal sites can be used to predict the risk of CNS relapse; the 2‐year rate of CNS relapse was high (34.5%) in patients with ≥3 extranodal sites.

Nowadays, CNS‐IPI and the number of extranodal sites are clinically valuable tools for predicting CNS relapse. However, these two models share the problem of low PPV (approximately 10%–15%) [11]. Similarly, elevated sIL‐2R levels, which were also identified as an independent risk factor for developing CNS relapse in our study, had a low PPV (25.7%). Since the use of elevated sIL‐2R levels improved the PPV of ≥3 extranodal sites from 35.3% to 71.4%, we proposed a predictive tool that combines the use of elevated sIL‐2R levels and number of extranodal sites for identification of patients at very high risk of CNS relapse.

Involvement of specific extranodal sites, such as the testis and kidney/adrenal glands, displays the strongest evidence of a high risk of CNS relapse in DLBCL cases [8, 14, 15, 16, 17]. Although involvement of the breast, uterus, paranasal sinuses, and bone have also been reported as risk factors for CNS relapse [33, 34, 35, 36, 37], involvement sites with high risk of CNS relapse vary among studies, owing to study design heterogeneity, including methods used to diagnose the presence of extranodal sites (using PET/CT or not), differences in tumor sites to be analyzed, and the years when the studies were conducted (in the pre‐ or postrituximab era). In this study, we analyzed DLBCL patients in the rituximab era and focused on detailed involvement sites defined by the Lugano 2014 criteria [27]. Since the incidence of CNS relapse in patients without specific involvement sites (the kidney/adrenal gland, testis, bone/bone marrow, paranasal sinus, and spleen) was very low, and involvement of any one of these five presented a very high NPV (99.0%), information concerning involvement sites might be beneficial in screening for likelihood of CNS relapse. However, it is insufficient to identify patients at high risk of CNS relapse owing to the low PPV (19.3%). Furthermore, rare involvement sites, such as the breast, uterus, and other sites, have not been sufficiently analyzed in this study because of our limited patient cohort. Therefore, to determine the anatomical factors that are associated with a very high risk of CNS relapse, there is a need for further investigations with larger patient cohorts.

The British Society of Hematology suggests the use of CNS prophylaxis in patients with (1) a high CNS‐IPI score (4 to 6), (2) three or more extranodal sites, or (3) involvement of the testis, kidney/adrenal gland, or intravascular sites [38]. Although there is no consensus regarding which methods are superior in preventing CNS relapse, two methods are used in clinical practice (IT‐MTX and intravenous high‐dose MTX [HD‐MTX]). Several reports indicate that IT is insufficient to prevent CNS relapse, whereas others have indicated that IT prolongs the time to CNS relapse [2, 4, 39, 40, 41, 42]. Conversely, intravenous HD‐MTX, another option, has been reported to reduce CNS relapse rates, while some studies did not report any efficacy [41, 43, 44, 45, 46]. In our study, although all of patients at high risk of CNS relapse did not receive CNS prophylaxis, IT prophylaxis may be insufficient to prevent CNS relapse. Of note, the numbers of patients with CNS relapse were not enough to evaluate the effectiveness of CNS prophylaxis in our study. Thus, further investigations, especially involving prospective studies, are needed to identify optimal methods to prevent CNS relapse.

This study had several limitations. First, the number of patients who developed CNS relapse was small owing to the single‐center retrospective nature of the study. Second, we did not evaluate the cell of origin, BCL2/MYC expression, and/or tumor translocation. Third, the decision to perform CNS prophylaxis was based on the treating physician's preference. Furthermore, it is inconclusive in assessing the benefits of CNS prophylaxis. Therefore, to confirm our data, there is a need for future prospective studies with a greater number of patients.

In conclusion, we proposed a new predictive tool to identify DLBCL patients at high risk of CNS relapse. This is a simple and clinically valuable tool for the prediction of CNS relapse in DLBCL cases, although additional validation studies are needed involving independent cohorts.

## CONFLICT OF INTEREST

Hiro Tatetsu has received honoraria from Ono Pharmaceutical, Chugai Pharmaceutical, Eisai, and patents, and royalties from Mesoblast. Kisato Nosaka has received consultancy fee, research funding, and honoraria from Kyowa Kirin and research funding from Chugai Pharmaceutical and honoraria from Celgene, Eisai, Meiji Seika Pharma, Janssen Pharmaceutical, Abbvie Inc. and Bristol Myers Squibb. Masao Matsuoka has received research funding from Chugai Pharmaceutical and Kyowa Kirin. Takafumi Shichijo, Yusuke Higuchi, Yoshitaka Kikukawa, Yoshitaka Inoue, Kosuke Toyoda, and Jun‐ichirou Yasunaga have no conflict of interest to disclose.

## AUTHOR CONTRIBUTIONS

*Conception and design*: Takafumi Shichijo and Hiro Tatetsu. *Provision of study materials or patients*: Takafumi Shichijo, Hiro Tatetsu, Kisato Nosaka, Yusuke Higuchi, Yoshitaka Kikukawa, Yoshitaka Inoue, Kosuke Toyoda, Jun‐ichirou Yasunaga, and Masao Matsuoka. *Collection and assembly of data*: Takafumi Shichijo and Hiro Tatetsu. *Data analysis*: Takafumi Shichijo and Hiro Tatetsu. *Interpretation of data*: Takafumi Shichijo, Hiro Tatetsu, Kisato Nosaka, Yusuke Higuchi, Yoshitaka Kikukawa, Yoshitaka Inoue, Kosuke Toyoda, Jun‐ichirou Yasunaga, and Masao Matsuoka. *Manuscript writing*: Takafumi Shichijo and Hiro Tatetsu. *Final approval of manuscript*: Takafumi Shichijo, Hiro Tatetsu, Kisato Nosaka, Yusuke Higuchi, Yoshitaka Kikukawa, Yoshitaka Inoue, Kosuke Toyoda, Jun‐ichirou Yasunaga, and Masao Matsuoka.

## Supporting information



Supporting Information
