# Supplementary material for: Predictive impact of soluble interleukin‐2 receptor and number of extranodal sites for identification of patients at very high risk of CNS relapse in diffuse large B‐cell lymphoma
Source: EJHaem. 2022 Feb 8;3(2):385–93. doi: 10.1002/jha2.393 (PMC9175809; doi:10.1002/jha2.393)
Supplement: Supplementary file 1 — Supporting Information [file JHA2-3-385-s001.docx]

**List of Supporting Information**

**Figure S1. ROC curve analysis of sIL-2R levels**

Abbreviations: ROC, receiver operating characteristic; sIL-2R, soluble interleukin-2 receptor

**Figure S2. Outcome of intrathecal chemotherapy for prophylaxis of CNS relapse**

Probability of overall survival in CNS-relapse patients treated with or without CNS prophylaxis.

Abbreviation: CNS, central nervous system

**Table S1. Incidence of CNS relapse in patients with or without CNS prophylaxis**

Abbreviation: CNS, central nervous system

**Supplementary Figure 1.**

**Supplementary Figure 2.**

Table S1. The incidence of CNS relapse in patients with or without CNS prophylaxis

|  | CNS prophylaxis | | No CNS prophylaxis | | *P-*value |
| --- | --- | --- | --- | --- | --- |
|  | Number of patients, n (%) | 2-year rate of CNS relapse | Number of patients, n (%) | 2-year rate of CNS relapse |  |
| CNS-IPI high risk | 11 (22.9) | 28.6 (8.0−74.2) | 37 (77.1) | 19.6 (9.3−38.6) | 0.979 |
| ≥3 Extranodal sites | 5 (29.4) | 50.0 (15.5−94.2) | 12 (70.6) | 27.8 (9.8−64.3) | 0.858 |
| Elevated sIL-2R | 7 (20.0) | 67.1 (22.3−94.2) | 28 (80.0) | 19.8 (8.7−41.3) | 0.241 |
| Kidney/adrenal gland | 5 (38.5) | 50.0 (15.5−94.2) | 8 (61.5) | 33.3 (9.6−80.5) | 0.956 |
| Bone/bone marrow | 11 (22.0) | 25.0 (6.6−70.2) | 39 (78.0) | 16.4 (7.7−32.9) | 0.442 |
| Testis | 9 (81.8) | 11.1 (1.6−56.7) | 2 (18.2) | 50.0 (9.0−99.4) | 0.142 |
| Spleen | 3 (17.6) | 33.3 (5.5−94.6) | 14 (82.4) | 22.6 (7.9−55.1) | 0.824 |
| Paranasal sinus | 10 (41.7) | 33.3 (12.2−71.8) | 14 (58.3) | 7.1 (1.0−40.9) | 0.550 |

Abbreviations: CNS, central nervous system; IPI, International Prognostic Index; LDH, lactate dehydrogenase; ULN, upper limit of normal; sIL-2R, soluble interleukin-2 receptor;
